# Stability of lactate dehydrogenase in plasma at different temperatures: post-analytical storage

**DOI:** 10.1515/almed-2020-0077

**Published:** 2020-10-07

**Authors:** Marta Gómez López, Neila Rodríguez Roca, Manuela Simón Velasco, María José Alcaide Martín, Antonio Buño Soto, Rubén Gómez Rioja

**Affiliations:** Laboratory Medicine Department, La Paz University Hospital, Madrid, Spain

**Keywords:** lactate dehydrogenase (LDH), post-analytical, stability

To the Editor,

Sample storage conditions are crucial to pre-analytical and post-analytical storage in clinical laboratories. Samples are routinely refrigerated during the post-analytical phase. Refrigeration reduces cellular metabolism and diminishes degradation reactions in a large number of analytes, although it may not be an optimal storage condition for other. Such is the case of lactate dehydrogenase (LDH), although consensus about the optimal temperature of storage for LDH remains elusive. In this context, a study was performed according to the protocol published by the Spanish Society for Laboratory Medicine (SEQC^ML^) [1] to determine the limit of stability for LDH in plasma stored both at room temperature (RT) and refrigerated.

In a preliminary study, determinations were performed at baseline and at the established analysis times, which coincided with usual post-analytical storage times in our laboratory. The maximum allowable difference (MAD) was established according to laboratory quality standards (6.39%, minimum systematic error calculated using the BV formula provided in the SEQC^ML^ database) [2].

For such purpose, the surplus of three lithium heparin plasma samples was employed. Each sample was split into three aliquots: a baseline aliquot was stored at −80 °C immediately after collection (baseline sample), whereas the other aliquots were stored at RT and at 4–8 °C. After 48 h, all aliquots were stored at −80 °C. Then, samples were thawed, homogenized and analyzed in the same run. Following IFCC guidelines, LDH activity was determined by the lactate:pyruvate method in a Dimension Vista 1500 analyzer (Siemens Healthineers). Determinations were performed in sextuplicate considering the ratio between MAD and the usual analytical imprecision in our laboratory (CV=2.97%).

Loss of stability was expressed as the percentage difference (%PD) among replicate measurements in each sample calculated by the formula %PD=T_time_−T_basal_/T_basal_*100. No significant variations were observed at room temperature (%PD < MAD). In contrast, a 12–15% loss exceeding MAD was observed in refrigerated samples (Table 1).

**Table 1:** Summary of preliminary study results.

	Patient 1			Patient 2			Patient 3		
	Baseline	Room temperature	Refrigerated	Baseline	Room temperature	Refrigerated	Baseline	Room temperature	Refrigerated
Mean	591.83	611.50	518.00	242.67	248.33	211.67	259.17	251.83	219.00
SD	9.83	16.47	3.22	3.20	5.16	3.44	1.47	1.33	3.52
%CV	1.66	2.69	0.62	1.32	2.08	1.63	0.57	0.53	1.61
%PD		3.32	−12.48		2.34	−12.77		−2.83	−15.50
MAD%		6.39%	6.39%		6.39%	6.39%		6.39%	6.39%

PD, percentage difference; MAD, maximum allowable difference.

An extended study was performed to calculate the limit of stability under refrigeration. For such purpose, 10 samples of plasma were split into seven aliquots that corresponded to each time studied: baseline, 12, 24, 36, 48, 60 and 72 h, which were stored at 4–8 °C. The aliquots that had been stored in a refrigerator were frozen at −80 °C at each time studied. After the established time, all aliquots were thawed, homogenized and analyzed in a single run in duplicate.

An instability equation (%PD=−0.14*time (h)) adjusted using the least squares method was designed. Pearson’s correlation coefficient (R) was 0.87 and a gradient far from 0 was obtained. This equation shows a daily loss of stability of 3.4%. In other words, for a MAD of 6.39%, the limit of stability was 45 h (Figure 1).

**Figure 1:**
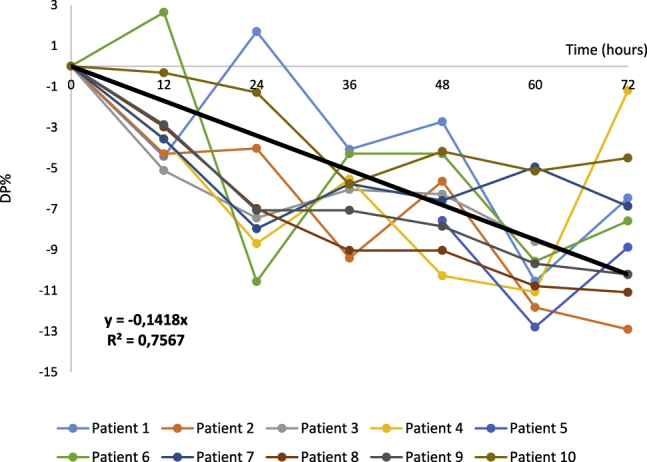
Graphic representation of the extended study.

The results of our study are consistent with the ones obtained by Shimizu Y, An B and Heins M [3], [4], [5], who reported that activity decreases in refrigerated samples, whereas it remains stable at RT. In contrast, Oddoze C [6] observed an increased activity. Limits of stability ranged from 1 to 4 days, which can be explained by different MAD criteria, which ranged from 3.53 to 7%.

The loss of stability of LDH under refrigeration could be caused by the different thermosensitivity of LDH isoenzymes. LDH-4 and 5 are especially labile and are likely to be the elements responsible for the loss of activity under refrigeration [4]. This could also explain the substantial between-subject variability observed. The different thermosensitivity of LDH isoenzymes may explain that the three patients included in the preliminary study exhibited a greater loss of stability than predicted by the extended model, which included a higher number of patients.

The information available about the stability of biochemical analytes is often confusing. Verification of stability limits in each laboratory guarantees that the limit of stability is adjusted to the specific conditions of each laboratory, such as the type of tube, temperature, light or shaking. The preliminary study proposed by SEQC^ML^ allows laboratories to confirm the validity of a usual stability limits, adapted to routine laboratory practice in a straightforward and cost-effective way. In case a potential loss of stability is detected, an extended study will allow to calculate an equation of instability that can be shared with other laboratories that work in similar conditions.

## Supplementary Material

Supplementary Material
