# Supplementary material for: Stability of lactate dehydrogenase in plasma at different temperatures: post-analytical storage
Source: Adv Lab Med. 2020 Oct 7;1(4):20200077. doi: 10.1515/almed-2020-0077 (PMC10197273; doi:10.1515/almed-2020-0077)
Supplement: Supplementary file 1 — Supplementary Material [file j_almed-2020-0077_suppl.doc]

**Annex**

Annex 1. Comprehensive preliminary study data obtained with Dimension Vista 1500.

|  | **Patient 1** | | |
| --- | --- | --- | --- |
|  | **Baseline** | **Room temperature** | **Refrigerated** |
| **Repeat 1** | 602 | 613 | 522 |
| **Repeat 2** | 601 | 627 | 519 |
| **Repeat 3** | 598 | 620 | 521 |
| **Repeat 4** | 588 | 599 | 515 |
| **Repeat 5** | 583 | 625 | 517 |
| **Repeat 6** | 579 | 585 | 514 |
| **Mean** | 591 .83 | 611 .50 | 518 .00 |
| **SD** | 9 .83 | 16 .47 | 3 .22 |
| **CV%** | 1 .66 | 2 .69 | 0 .62 |
| **%PD** |  | 3 .32 | -12 .48 |
| **MAD%** |  | 6 .39% | 6 .39% |

|  | **Patient 2** | | |
| --- | --- | --- | --- |
|  | **Baseline** | **Room temperature** | **Refrigerated** |
| **Repeat 1** | 248 | 257 | 217 |
| **Repeat 2** | 245 | 245 | 214 |
| **Repeat 3** | 240 | 251 | 211 |
| **Repeat 4** | 241 | 249 | 211 |
| **Repeat 5** | 240 | 245 | 210 |
| **Repeat 6** | 242 | 243 | 207 |
| **Media** | 242 .67 | 248 .33 | 211 .67 |
| **SD** | 3 .20 | 5 .16 | 3 .44 |
| **%CV** | 1 .32 | 2 .08 | 1 .63 |
| **%PD** |  | 2 .34 | -12 .77 |
| **MAD%** |  | 6 .39% | 6 .39% |

|  |  | **Patient 3** | |
| --- | --- | --- | --- |
|  | **Baseline** | **Room temperature** | **Refrigerated** |
| **Repeat 1** | 260 | 250 | 219 |
| **Repeat 2** | 261 | 252 | 223 |
| **Repeat 3** | 258 | 251 | 222 |
| **Repeat 4** | 260 | 252 | 218 |
| **Repeat 5** | 257 | 252 | 219 |
| **Repeat 6** | 259 | 254 | 213 |
| **Media** | 259 .17 | 251 .83 | 219 .00 |
| **SD** | 1 .47 | 1 .33 | 3 .52 |
| **%CV** | 0 .57 | 0 .53 | 1 .61 |
| **%PD** |  | -2 .83 | -15 .50 |
| **MAD%** |  | 6 .39% | 6 .39% |

Annex 2. Comprehensive preliminary study data obtained with Dimension Vista 1500.

|  | | **Baseline** | **12 hours** | **24 hours** | **36 hours** | **48 hours** | **60 hours** | **72 hours** |
| --- | --- | --- | --- | --- | --- | --- | --- | --- |
| **Patient 1** | **Repeat 1** | 147 | 142 | 151 | 139 | 141 | 133 | 136 |
|  | **Repeat 2** | 147 | 139 | 148 | 143 | 145 | 130 | 139 |
|  | **Mean** | 147 | 140 .5 | 149 .5 | 141 | 143 | 131 .5 | 137 .5 |
|  | **SD** | 0 .00 | 2 .12 | 2 .12 | 2 .83 | 2 .83 | 2 .12 | 2 .12 |
|  | **%CV** | 0 .00 | 1 .51 | 1 .42 | 2 .01 | 1 .98 | 1 .61 | 1 .54 |
| **Patient 2** | **Repeat 1** | 185 | 178 | 178 | 167 | 175 | 161 | 160 |
|  | **Repeat 2** | 187 | 178 | 179 | 170 | 176 | 167 | 164 |
|  | **Mean** | 186 | 178 | 178 .5 | 168 .5 | 175 .5 | 164 | 162 |
|  | **SD** | 1 .41 | 0 .00 | 0 .71 | 2 .12 | 0 .71 | 4 .24 | 2 .83 |
|  | **%CV** | 0 .76 | 0 .00 | 0 .40 | 1 .26 | 0 .40 | 2 .59 | 1 .75 |
| **Patient 3** | **Repeat 1** | 213 | 205 | 195 | 203 | 201 | 195 | 193 |
|  | **Repeat 2** | 217 | 203 | 203 | 201 | 202 | 198 | 193 |
|  | **Mean** | 215 | 204 | 199 | 202 | 201 .5 | 196 .5 | 193 |
|  | **SD** | 2 .83 | 1 .41 | 5 .66 | 1 .41 | 0 .71 | 2 .12 | 0 .00 |
|  | **%CV** | 1 .32 | 0 .69 | 2 .84 | 0 .70 | 0 .35 | 1 .08 | 0 .00 |
| **Patient 4** | **Repeat 1** | 126 | 122 | 115 | 122 | 113 | 111 | 125 |
|  | **Repeat 2** | 127 | 122 | 116 | 117 | 114 | 114 | 125 |
|  | **Mean** | 126 .5 | 122 | 115 .5 | 119 .5 | 113 .5 | 112 .5 | 125 |
|  | **SD** | 0 .71 | 0 .00 | 0 .71 | 3 .54 | 0 .71 | 2 .12 | 0 .00 |
|  | **%CV** | 0 .56 | 0 .00 | 0 .61 | 2 .96 | 0 .62 | 1 .89 | 0 .00 |
| **Patient 5** | **Repeat 1** | 191 | 189 | 179 | 178 | 179 | 168 | 177 |
|  | **Repeat 2** | 192 | 183 | 177 | 177 | 175 | 166 | 172 |
|  | **Mean** | 191 .5 | 186 | 178 | 177 .5 | 177 | 167 | 174 .5 |
|  | **SD** | 0 .71 | 4 .24 | 1 .41 | 0 .71 | 2 .83 | 1 .41 | 3 .54 |
|  | **%CV** | 0 .37 | 2 .28 | 0 .79 | 0 .40 | 1 .60 | 0 .85 | 2 .03 |
| **Patient 6** | **Repeat 1** | 148 | 157 | 137 | 145 | 147 | 137 | 144 |
|  | **Repeat 2** | 155 | 154 | 134 | 145 | 143 | 137 | 136 |
|  | **Mean** | 151 .5 | 155 .5 | 135 .5 | 145 | 145 | 137 | 140 |
|  | **SD** | 4 .95 | 2 .12 | 2 .12 | 0 .00 | 2 .83 | 0 .00 | 5 .66 |
|  | **%CV** | 3 .27 | 1 .36 | 1 .57 | 0 .00 | 1 .95 | 0 .00 | 4 .04 |
| **Patient 7** | **Repeat 1** | 178 | 174 | 171 | 173 | 168 | 170 | 169 |
|  | **Repeat 2** | 186 | 177 | 164 | 170 | 172 | 176 | 170 |
|  | **Mean** | 182 | 175 .5 | 167 .5 | 171 .5 | 170 | 173 | 169 .5 |
|  | **SD** | 5 .66 | 2 .12 | 4 .95 | 2 .12 | 2 .83 | 4 .24 | 0 .71 |
|  | **%CV** | 3 .11 | 1 .21 | 2 .96 | 1 .24 | 1 .66 | 2 .45 | 0 .42 |
| **Patient 8** | **Repeat 1** | 494 | 486 | 448 | 442 | 441 | 436 | 431 |
|  | **Repeat 2** | 480 | 459 | 458 | 444 | 445 | 433 | 435 |
|  | **Mean** | 487 | 472 .5 | 453 | 443 | 443 | 434 .5 | 433 |
|  | **SD** | 9 .90 | 19 .09 | 7 .07 | 1 .41 | 2 .83 | 2 .12 | 2 .83 |
|  | **%CV** | 2 .03 | 4 .04 | 1 .56 | 0 .32 | 0 .64 | 0 .49 | 0 .65 |
| **Patient 9** | **Repeat 1** | 191 | 183 | 179 | 176 | 178 | 171 | 172 |
|  | **Repeat 2** | 191 | 188 | 176 | 179 | 174 | 174 | 171 |
|  | **Mean** | 191 | 185 .5 | 177 .5 | 177 .5 | 176 | 172 .5 | 171 .5 |
|  | **SD** | 0 .00 | 3 .54 | 2 .12 | 2 .12 | 2 .83 | 2 .12 | 0 .71 |
|  | **%CV** | 0 .00 | 1 .91 | 1 .20 | 1 .20 | 1 .61 | 1 .23 | 0 .41 |
| **Patient 10** | **Repeat 1** | 154 | 153 | 154 | 145 | 151 | 147 | 147 |
|  | **Repeat 2** | 157 | 157 | 153 | 148 | 147 | 148 | 150 |
|  | **Mean** | 155 .5 | 155 | 153 .5 | 146 .5 | 149 | 147 .5 | 148 .5 |
|  | **SD** | 2 .12 | 2 .83 | 0 .71 | 2 .12 | 2 .83 | 0 .71 | 2 .12 |
|  | **CV%** | 1 .36 | 1 .82 | 0 .46 | 1 .45 | 1 .90 | 0 .48 | 1 .43 |
